# Impact of Total Neoadjuvant Therapy vs. Standard Chemoradiotherapy in Locally Advanced Rectal Cancer: A Systematic Review and Meta-Analysis of Randomized Trials

**DOI:** 10.3390/cancers12123655

**Published:** 2020-12-05

**Authors:** Maria C. Riesco-Martinez, Carlos Fernandez-Martos, Cristina Gravalos-Castro, Paula Espinosa-Olarte, Anna La Salvia, Luis Robles-Diaz, Andrea Modrego-Sanchez, Rocio Garcia-Carbonero

**Affiliations:** 1 Medical Oncology Department, Hospital Universitario 12 de Octubre, UCM, 28041 Madrid, Spain; cristina.gravalos@salud.madrid.org (C.G.-C.); paula.espinosa@salud.madrid.org (P.E.-O.); alasalvi@ucm.es (A.L.S.); luis.robles@salud.madrid.org (L.R.-D.); andrea.modrego@salud.madrid.org (A.M.-S.); rgcarbonero@salud.madrid.org (R.G.-C.); 2 Laboratorio de Oncologia Clinico-Traslacional, Instituto de Investigacion Sanitaria Hospital 12 de Octubre (*imas12*), CNIO, CIBERONC, 28041 Madrid, Spain; 3 Medical Oncology Department, Initia Oncology, Hospital Quironsalud, 46010 Valencia, Spain; carlosfmartos@initiaoncologia.com

**Keywords:** meta-analysis, rectal cancer, neoadjuvant therapy, chemotherapy, treatment

## Abstract

**Simple Summary:**

Multimodality therapy is the standard of care for patients with locally advanced rectal cancer (LARC). The optimal treatment sequence is, however, a matter of debate. Neoadjuvant radiotherapy with concurrent fluoropyrimidines followed by surgery and adjuvant chemotherapy has been the standard treatment for the past years. Alternative therapeutic strategies such as total neoadjuvant treatment (TNT) are gaining momentum, although results from individual clinical trials are not conclusive regarding its impact on survival. In this context, we aimed to systematically review available evidence from randomized trials comparing different sequencing strategies. The results from our meta-analysis show that TNT not only provides increased complete pathological response rates, but also improves disease-free and overall survival at 3 years compared to standard neoadjuvant chemoradiotherapy, with no substantial increase in severe adverse events. These results support the use of induction or consolidation chemotherapy before surgery in LARC and TNT as a valuable treatment strategy in these patients.

**Abstract:**

Multimodality treatment is a standard of care for LARC, but the optimal sequencing of the treatment modalities remains unclear. Several randomized clinical trials (RCTs) compared total neoadjuvant treatment (TNT) vs. standard neoadjuvant chemoradiotherapy (CRT) with inconsistent results. A systematic review and meta-analysis was performed to evaluate the efficacy of TNT in terms of complete pathological response (pCR) rate, disease-free and overall survival vs. standard CRT in LARC. A systematic search was performed through MEDLINE, EMBASE, Cochrane Central Register of Controlled Trials and meeting abstracts up to May 2020. RCTs comparing CRT vs. TNT followed by surgery in LARC were eligible for the study. Study selection and data extraction were done following PRISMA guidelines by two independent reviewers. The Mantel–Haenzel method was used to obtain a fixed-effects model of pooled odds or hazard ratios for the main outcomes. Eight RCTs, including 2301 patients, met the eligibility criteria. TNT significantly improved pCR rate (OR = 1.99, 95% confidence interval (CI) 1.59–2.49; *p* < 0.001), 3-year disease-free-survival (DFS) (HR = 0.82, 95%CI 0.71–0.95; *p* = 0.01) and 3-year overall survival (OS) (hazard ratio (HR) = 0.81, *p* = 0.04). Grade 3–4 adverse events were not significantly different in both strategies (OR = 1.58; *p* = 0.14). An improved pCR rate was documented regardless of the type of radiotherapy administered (long vs. short fractionation schedules). No significant heterogeneity was found. The results of this meta-analysis show that TNT improves pCR and survival rates vs. standard preoperative CRT in patients with LARC. TNT may become a new standard of care in LARC, although longer follow-up is needed to properly assess its long-term impact on survival.

## 1. Introduction

Over the past decades, the standard of care for locally advanced rectal cancer (LARC) has remarkably evolved. The improvement of surgical techniques and the addition of neoadjuvant chemo-radiotherapy (CRT) or short-course radiotherapy (SCRT) have reduced the 5-year locoregional recurrence rate to 5–8% [1,2,3]. Despite these improvements, ~30% of patients still develop distant metastasis, which remains the leading cause of rectal-cancer-related death [4,5]. This has shifted the focus into the role of systemic therapy and its optimal timing in order to decrease distant failure.

The optimal succession of strategies to administer multimodality treatment is still a matter of debate [6]. The most widely adopted strategy consists of CRT with concurrent fluoropyrimidines, followed by surgery and adjuvant chemotherapy (CT). However, the benefit of post-surgical CT has not been definitively proven and treatment compliance in this setting is rather poor [3]. Indeed, about 25% of patients randomized to receive adjuvant CT never actually started treatment and only ~50% received the planned dose [3]. The total neoadjuvant therapy (TNT) approach, in which all CT and RT are administered preoperatively, has been an active area of research in recent years. TNT consists of the addition of induction or consolidation CT before or after neoadjuvant CRT or SCRT, followed by surgery. Both provide several potential advantages including improved treatment tolerance and compliance, early treatment of micrometastases, and greater rates of complete tumor response, thereby overcoming some of the limitations from adjuvant CT. On the other hand, this may be carried out at the risk of an increased toxicity and overtreatment for the patient. Only a few randomized clinical trials (RCTs) have properly assessed the role of this strategy compared to standard neoadjuvant CRT with inconsistent results. The small size of some of these studies, the different TNT regimens used and the inclusion of a low-risk population may have underestimated the real effect of this approach. 

To help elucidate the role of TNT in this context, we conducted a systematic review and meta-analysis of RCTs assessing the addition of induction or consolidation CT to standard preoperative CRT in patients with stage II–III rectal adenocarcinoma.

## 2. Results

### 2.1. Literature Search Results and Study Characteristics

**Figure 1:**
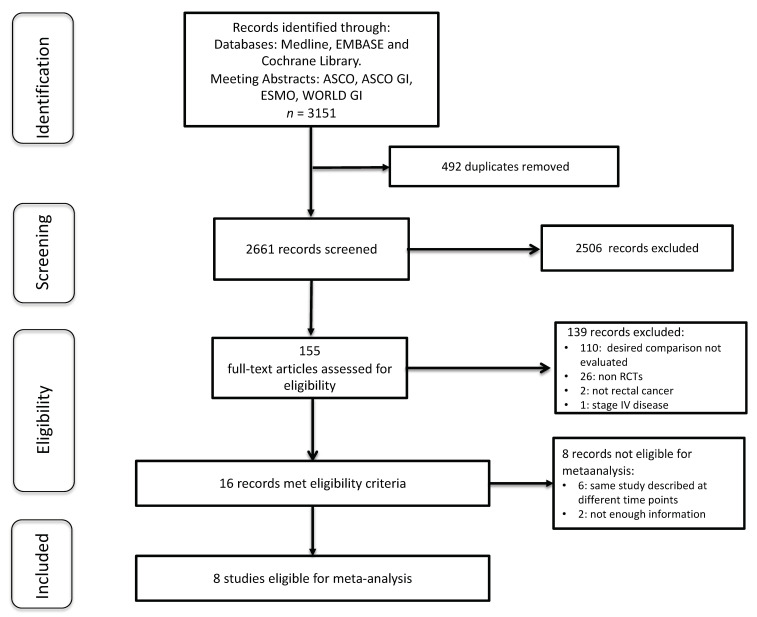
Flow chart showing study selection.

A total of 3151 articles were identified through a literature search and meeting abstracts (Text S1). After the deduplication and exclusion of 2645 non-relevant records, 16 potentially eligible RCTs were selected. Among them, five were updates of prior published studies, one was a secondary analysis of an already included RCT, and two were discarded for not having enough information on the main outcome. Hence, eight different RCTs comparing TNT to standard CRT were included in the present meta-analysis [7,8,9,10,11,12,13,14]. (Figure 1).

A total of 2301 patients were included, of whom 1131 (49.2%) received standard neoadjuvant CRT and 1170 (50.8%) TNT. The main trials characteristics are summarized in Table 1. Patient characteristics were overall well balanced among study arms in all studies (Table 2). Only the GCR-3 trial showed a higher proportion of T4 tumors in the experimental arm (12 vs. 5.8%) [7]. Four RCTs used induction CT (*n* = 717) and 4 (*n* = 1584) consolidation CT as part of the TNT strategy. In the experimental arm, CRT and SCRT were used in six (*n* = 874) and two (*n* = 1427) RCTs, respectively. All studies but one [10], which used 5-FU monotherapy, administered polychemotherapy as part of the TNT regimen. FOLFOX(fluorouracil, folinic acid and oxaliplatin)/XELOX(capecitabine and oxaliplatin) were the most frequent regimens used. Duration of CT in the TNT group ranged from 1.5 to 4.5 months. Five trials reported compliance data, with an average of 92% of patients completing treatment. Only two trials [7,13] presented data on adjuvant treatment compliance (54% and 75%, respectively). Surgical outcomes from included trials are depicted in Table S1.

### 2.2. Study Quality Assessment 

A list of biases is summarized in Figures S1 and S2. All included studies were randomized and followed intention-to-treat analysis for the primary endpoints. Two of the studies [13,14] were published only in abstract form. All trials reported pCR rate, but only four provided data for disease-free-survival (DFS) and overall survival (OS). Heterogeneity was present in some pairwise treatment comparisons based on I^2^; however, the studies were comparable in terms of patient characteristics and outcomes. No evidence of publication bias was observed (Figure S3)

### 2.3. Pathological Complete Response Rates

**Figure 2:**
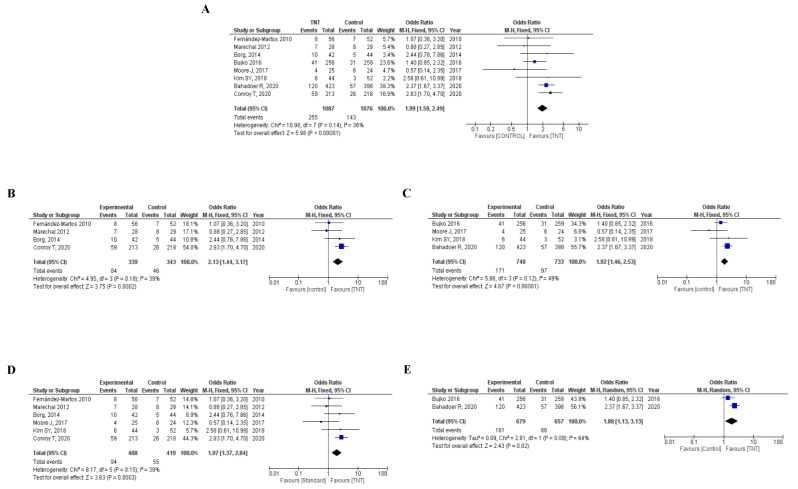
(**A**) Forest plot for pathologic complete response of TNT vs. standard treatment. Forest plot for pathologic complete response of TNT vs. standard treatment using: (**B**) induction chemotherapy in the experimental arm; (**C**) consolidation chemotherapy in the experimental arm; (**D**) long-course chemo-radiotherapy in the experimental arm; (**E**) short-course radiotherapy in the experimental arm. CI: confidence interval, OR: odd ratio, TNT: total neoadjuvant therapy.

Overall, including the eight RCTs selected, 398 of 2163 patients (18.4%) achieved a pCR after neoadjuvant therapy, 255 of 1087 (23.5%) in the TNT arm and 143 of 1076 (13.3%) in the standard arm. (OR 1.99, 95% confidence interval (CI) 1.59–2.49, *p* < 0.001). No significant heterogeneity was found (I^2^ = 36%, *p* = 0.14). (Figure 2A). The sensibility analysis performed showed stability of the pooled OR with marginal fluctuations when excluding each study at a time (Table S1). 

In the four RCTs that used induction chemotherapy [7,8,9,13], 130 of 682 (19.1%) randomized patients achieved a pCR, 84 of 339 (24.8%) in the TNT group and 46 of 343 (13.4%) in the standard arm (OR 2.13, 95% CI 1.44–3.17, *p* < 0.001; I^2^ = 39%, *p* = 0.18). In the four trials that used consolidation CT in the TNT arm [10,11,12,14], 268 patients of 1481 (18.1%) achieved a pCR, 171 of 748 (22.9%) in the experimental arm and 97 of 733 (13.2%) in the standard group. (OR 1.92, 95% CI 1.46–2.53, *p*< 0.001; I^2^ = 49%, *p* = 0.12). (Figure 2B,C). 

Six trials used CRT in the experimental and control arms (*n* = 827) [7,8,9,11,12,13]. In these RCTs, 149 of 827 (18%) randomized patients achieved a pCR, 94 of 408 (23.0%) in the TNT arm and 55 of 419 (13.1%) in the standard arm (OR 1.97, 95% CI 1.37–2.84, *p*< 0.001; I^2^ = 39%, *p* = 0.15). Two RCTs used SCRT in the TNT arm [10,14]. Globally, 249 of 1336 (18.6%) patients achieved a pCR, 161 of 679 (23.7%) in the experimental group and 88 of 657 (13.4%) in the standard arm (OR 1.88, 95% CI 1.13–3.13, *p* = 0.02; I^2^ = 64%, *p* = 0.09) (Figure 2D,E). Tables S1–S4 display the sensitivity analysis.

### 2.4. Disease-Free and Overall Survival

**Figure 3:**
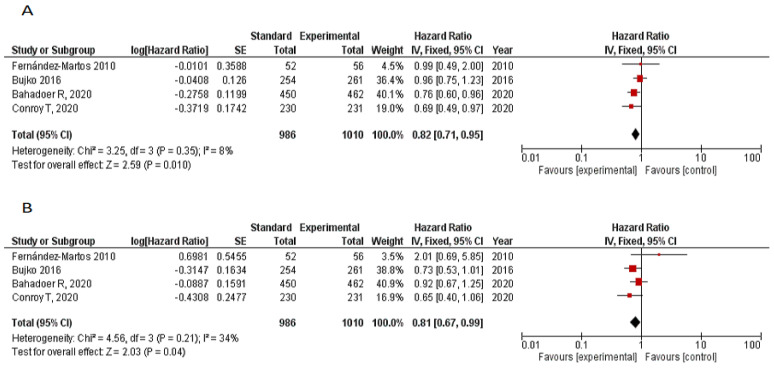
(**A**) Forest plot for 3-year DFS TNT vs. CRT. (**B**) Forest plot for 3-year OS TNT vs. CRT.

Four RCTs reported survival outcomes [7,10,13,14]. Median follow-up is summarized in Table 1. Meta-analysis of DFS included 1996 patients and 1010 and 986 patients in the TNT and standard arms, respectively. Pooled hazard ratio (HR) with fixed effects model for progression showed a significant difference in favor of the experimental group (HR 0.82, 95% CI 0.71–0.95, *p* = 0.01), with no significant heterogeneity (I^2^ = 8%, *p* = 0.35). OS analysis included 1996 patients. The pooled HR with fixed effects model for OS was 0.81 (95% CI 0.67–0.99, *p* = 0.04; I^2^ = 34%, *p* = 0.21) favoring the TNT arm (Figure 3A,B). 

### 2.5. R0 Resection Rates

Six RCTs reported data on the type of resection accomplished [7,8,10,11,12,14]. In 1404 of 1646 patients (85.3%), an R0 resection was achieved, 721 of 836 (86.2%) in the experimental arm and 683 of 810 (84.3%) in the standard group. No significant differences were observed between groups (OR 1.18, 95% CI 0.89–1.55, *p* = 0.25; I^2^ = 25%, *p* = 0.24). Tumor regression grade was only reported in three RCTs, including 261 patients [7,8,12]. (Figure S4A,B).

### 2.6. Grade 3–4 Adverse Events

**Figure 4:**
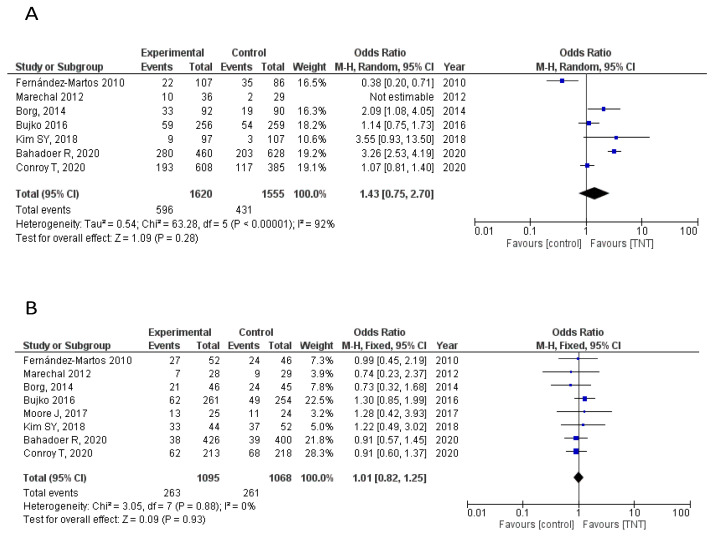
(**A**) Forest plot for G3–4 adverse events. (**B**) Forest plot for G3–4 adverse events due to surgical complications.

Seven RCTs provided data on Grade 3–4 adverse events (AEs) [7,8,9,10,12,13,14]. Overall, Grade 3–4 AEs were considered as any AE occurring during the course of neoadjuvant or adjuvant treatment. No statistically significant differences were found between the experimental and standard arms in overall G3–4 AEs (OR = 1.43, 95% CI 0.75–2.70). Specifically, data on any post-surgical complications occurring up to 30 days after surgery were also analyzed. No statistically significant differences between arms was found either (OR = 1.01, 95% CI 0.82–1.25) (Figure 4A,B).

## 3. Discussion

Trimodality therapy with neoadjuvant CRT, total mesorectal excision (TME) and adjuvant CT has been the standard of care in LARC for the past decades. However, the contribution of adjuvant CT in this strategy is questionable [15,16], and the high systemic treatment failure remains a matter of concern. In this context, administering CT at earlier stages of the treatment strategy as in TNT has gained momentum as it can overcome some of the caveats of the conventional approach, improving outcomes and allowing for more conservative surgical procedures or even avoiding them within careful watch-and-wait strategies. 

This systematic review and meta-analysis aimed to elucidate the role of TNT compared to standard neoadjuvant therapy in LARC. The results of our study, including eight RCTs and 2301 patients, demonstrate a significant absolute 10.2% increased pCR rate (23.5% vs. 13.3%) with TNT compared to standard neoadjuvant therapy (OR 1.99, *p* < 0.001). The beneficial effect of TNT was maintained when either the induction or consolidation CT, or long- versus short-fractionation RT studies, were analyzed separately. The 23.5% pCR rate calculated for TNT in the present meta-analysis was remarkably high, especially considering the great proportion of patients with high-risk features included in these trials such as T4, N2 or circumferential resection margin involvement. Moreover, it was superior to the pCR rates previously reported with other strategies that also aimed to intensify treatment preoperatively, such as the addition of oxaliplatin to CRT that showed a 19% pCR in a pooled analysis [17]. 

One relevant factor to take into consideration when evaluating pCR is the interval from treatment completion to surgery. It is known that increased pCR rates are achieved when patients undergo surgery 6–8 weeks after CRT completion compared to those operated within 2 weeks [18]. However, the optimal time interval from neoadjuvant therapy to surgery remains unclear, with randomized trials showing conflicting results and European and American guidelines providing no specific recommendation with a suggested 4-to-12-week interval from neoadjuvant treatment. A recent meta-analysis including four RCTs showed that a minimum 8-week interval, compared to less than 8 weeks interval, was associated to increased pCR rates [19]. All studies included in our meta-analysis reported data on the time interval to surgery. Six to eight weeks was the most common time frame in which surgery was carried out (75%, 6/8 RCTs). In the other two trials [9,10], 11–12 weeks was the preferred waiting time to surgery. Therefore, and given the consistency among studies, it is unlikely that the time interval after neoadjuvant treatment may have biased the results of our study. 

pCR is often used as a surrogate marker of favorable oncological outcome [20,21] and it is a primary or secondary endpoint in most LARC trials. Indeed, our meta-analysis showed a significant improvement in 3-year DFS and OS favoring the TNT arm compared to standard neoadjuvant therapy, with an 18% decrease in the risk of recurrence (HR 0.82, 95% CI 0.71–0.95, *p* = 0.01) and a 19% decrease in the risk of death (HR 0.81, 95% CI 0.67–0.99, *p* = 0.04).

Additionally, these significant improvements in oncological outcomes were not obtained at the expense of increased toxicity. In fact, no significant differences in Grade 3–4 AEs were observed between the TNT and standard CRT (HR = 1.45, 95% CI 0.75–2.70, *p* = 0.28) and no relevant differences in post-surgical mortality was observed either (Table S1). Although reports on quality of life and functional outcome in these studies are scarce, no significant differences were seen in the reported QoL analysis from the recently presented PRODIGE-23 and RAPIDO trials [13,14] with no statistically significant differences found in low anterior resection syndrome (LARS) score among patients undergoing standard vs. experimental treatment in the RAPIDO trial (*p* = 0.19). Moreover, although in all trials included in this meta-analysis patients underwent TME resection per protocol, the higher pCR rates achieved with TNT facilitate the option of a non-operative management for responders. In fact, a growing amount of evidence questions the added value of TME in patients achieving a pCR. Recently, the results from the Organ Preservation in Rectal Adenocarcinoma (OPRA) trial evaluating this relevant matter have been presented [22]. A total of 324 patients with distal rectal cancer were randomized to two possible TNT strategies, consolidation vs. induction oxaliplatin-based chemotherapy plus CRT. Patients achieving a complete clinical response after treatment underwent a watch and wait approach, while TME was performed for non-responders. A non-operative management was performed in ~50% of patients (43% and 59% of those receiving induction and consolidation chemotherapy, respectively), with a 3-year DFS of 77% and 78% for the induction and consolidation strategies, respectively, superior to the historical controls. Therefore, although longer follow-up is needed, the increased pCR rates achieved with TNT strategies provide the potential advantage of not only improving survival outcomes, but also favoring organ preservation options which may clearly positively impact on the quality of life of these patients. 

It should be noted that, despite most trials allowing the inclusion of rectal cancer patients regardless of their location, the vast majority of included patients presented mid or low rectal cancer. Indeed, only 8.3% of patients with available data on tumor location had upper rectal cancer, although two trials did not report specific information on this important matter [11,12], and one trial [7] provided only the number of patients with low rectal location. This raises the question of whether these results may apply to patients with upper rectal cancer, as data on this particular tumor site are scarce. Moreover, the use of radiotherapy in this subset of patients has been questioned over the past years, since locoregional recurrence rates with TME alone are low and significant toxicity is associated with this additional treatment [23,24]. In fact, in the seminal German Rectal Cancer trial, while local recurrence at 5 years was 10.1% in low-third LARC, it was only 2.7% for tumors located > 10 cm from the anal verge [25]. Therefore, there is currently solid evidence supporting the superiority of TNT over standard neoadjuvant therapy for low and mid rectal tumors, whereas this strategy may be questionable for tumors located in the upper rectum. The current challenge nowadays is to better tailor treatment options and identify approaches that maintain or improve oncologic outcomes while minimizing morbidity.

Some limitations of the present study should be considered in the interpretation of our findings. Only four RCTs (*n* = 1996) provided data on survival [7,10,13,14], and, except for the GCR-3 study (median follow-up of 69 months), most trials still have a relatively short follow-up. This limits the power to detect a true effect and limits an adequate assessment of long-term outcomes. Moreover, relevant disparity in the regimens used as TNT was found among studies. Five studies provided neoadjuvant CT for less than 3 months [8,9,10,11,12] with one of them using only monotherapy as part of the TNT strategy [11]. Additionally, only two trials reported information on adjuvant therapy [7,13]. Finally, two studies have only been reported in abstract form [13,14]; the full manuscript publication of these shall provide valuable additional details for further analysis. Nevertheless, these limitations should not influence the overall interpretation of our results that provide valuable information on the debated role of TNT strategy in LARC. These findings give updated estimates on the magnitude of benefit of the TNT strategy providing solid evidence that this approach does not only increase pCR but also has a significant impact on survival in these patients. 

In summary, and to the best of our knowledge, our study is the largest meta-analysis on RCTs comparing TNT with standard neoadjuvant treatment in LARC and the first to demonstrate a significant impact on survival based on controlled data. A previous meta-analysis [26] included 28 studies, but the majority were retrospective and non-controlled trials. Only five were RCTs and just two reported survival outcomes. This pooled analysis of non-randomized trials showed improved pCR, DFS and OS rates favoring the TNT arm, consistent with our results. 

## 4. Materials and Methods

This systematic review and meta-analysis was performed in accordance with the Preferred Reported Items for Systematic Reviews and Meta-analyses (PRISMA) guidelines [27].

### 4.1. Search Strategy and Study Identification

Eligible studies were identified through a systematic review of published literature in Medline, EMBASE and Central Cochrane databases. Bibliographic references of relevant trials were also reviewed. No language or date restriction were placed up to May 2020. The search strategy was done using specific keywords and free text terms combined with Boolean operators (Text-S1). A manual search of conference proceedings from the American Society of Clinical Oncology (ASCO), European Society of Medical Oncology (ESMO) and Gastrointestinal Cancer symposiums between 2015 and 2020 was also performed to identify relevant unpublished studies. Relevant articles were cross-referenced to confirm that all possible pertinent records were identified. Two authors (MCR, PEO) independently screened titles and abstracts from identified studies to assess compliance with eligibility criteria. Disagreements were resolved by discussion to reach consensus or by the senior author (RGC). Full text screening was completed in duplicate to determine the final list of studies for inclusion.

### 4.2. Selection Criteria and Data Extraction

To be included in the present meta-analysis, eligible studies had to meet all of the following inclusion criteria: (1) stage II–III rectal cancer; (2) phase II or III RCTs; (3) RCTs including rectal cancer patients who received TNT in the experimental arm and standard CRT in the control arm; (4) sufficient information on patient characteristics, study design, and outcomes. 

Principal exclusion criteria were: (1) non-RCTs; (2) studies including TNT in both arms of the study; (3) stage IV rectal cancer; (4) overlapping publications. 

Two independent reviewers (MCR and PEO) screened titles and abstracts from the studies identified in the literature search to assess the compliance of the selection criteria. Disagreements were resolved by discussion or a third reviewer (RGC) to reach consensus. Full text screening was completed in duplicate to determine the final list of studies for inclusion. 

The following variables were extracted from all included RCTs if available: year of publication, study design, number of randomized patients, treatment regimen on each arm, complete pathological response rate (pCR; ypT0N0), disease free survival (DFS), overall survival (OS), R0 resection rate, tumor regression grade (TRG) rate, treatment compliance, grade 3–4 adverse events (G3–4AEs) and median follow-up in the standard and experimental arm.

### 4.3. Statistical Analysis

The primary objective was to compare the activity of TNT vs. standard neoadjuvant CRT for LARC in terms of pCR. Secondary objectives were DFS, OS, R0 resection rate, TRG rate and G3–4 AEs.

Descriptive statistics were used to summarize study and patients’ characteristics. Odds (OR) and hazard ratios (HR) and a 95% confidence interval (CI) were used when appropriate to calculate the effect of TNT vs. CRT for the main outcomes. The Mantel–Haenzel method [28] was used to obtain a fixed-effects model of pooled ORs. All tests and CIs were two-sided. Pooled ORs and HRs were considered statistically significant with a *p* value of <0.05. 

Heterogeneity between studies was assessed using Higgins I^2^ statistic [29]. In the presence of significant heterogeneity (I^2^ > 50%), the DerSimonian–Laird method was selected using the random effects model [30]. Risk of publication bias was assessed by visual inspection of funnel plots [31,32]. Quality of studies was independently assessed by two authors (MRC, PEO) using the Cochrane risk of bias tool [33]. Sensitivity analyses were conducted by recalculating pooled OR and HR estimates after exclusion of each individual study. Prespecified subgroup analysis was completed according to the type of CT in the TNT strategy (induction/consolidation) and radiotherapy fractionation (short/long course). RevMan 5.3 statistics was used for statistical analysis and forest plots’ generation. 

## 5. Conclusions

Compared with standard neoadjuvant therapy, our meta-analysis showed that TNT was associated with significantly increased pCR rates, DFS and OS in LARC with no significant increase in severe adverse events. These findings suggest that TNT may be considered a safe and more effective treatment option for patients with LARC. Longer follow-up is needed, however, to elucidate its impact on long-term patient outcomes in this setting. Additional controlled trials will be needed to address the optimal CT and RT regimens and schedules within the TNT strategy to further improve the fate of patients with LARC.

## Figures and Tables

**Table 1:** Main characteristics of randomized controlled trials included.

Study	Year	Study Design	*N*	Treatment Arms	RT Dose (Gys)	*N*	pCR	DFS (%)	HR (95% CI)	OS (%)	HR (95% CI)	Median Follow-Up (m)
Fernandez-Martos C (GCR-3) NCT-00421824.	2010	Randomized phase II	108	• CRT→Sx→XELOX • XELOX x 4→CRT→Sx	50.4	52 56	13.5 14.3	64 62	0.99	78 75	2.01 (0.69–5.85)	69
Marechal R EudraCT 2006-006646-34	2012	Randomized phase II	57	• CRT(5FU)→Sx • FOLFOX x 2→CRT(5FU)→Sx	45	28 29	28 25	NR	-	NR	-	NR
Borg C (INOVA) NCT 00865189	2014	Randomized phase II	91	• CRT (5FU-Bev)→Sx • FOLFOX+Bevx2→CRT (5FU-Bev)→Sx	45	45 46	11.4 23.8	NR	-	NR	-	NR
Bujko K NCT-00833131	2016	Phase III	515	• CRT→ Sx • SCRT→FOLFOX x3→Sx	50.4 25	254 261	12 16	52 53	0.96 (0.75–1.24)	65 73	0.73 (0.53–1.01)	35
Moore J (WAIT) ACTRN-12611000339954	2017	Randomized phase II	49	• CRT→Sx • CRT→5FU x 3→Sx	45 + 5.4 Boost	24 25	25 16	NR	-	NR	-	NR
Kim SY NCT-01952951	2018	Randomized phase II	108	• CRT→Sx • CRT→XELOX x 2→Sx	50.4	55 53	5.8 13.6	NR	-	NR	-	NR
Conroy T PRODIGE-23 NCT-01804790	2020	Phase III	461	• CRT→Sx→ FOLFOX x 12 • FOLFIRINOX x6→ CRT→Sx→FOLFOX x 6	50.4	230 231	12.1 27.8	68.5 75.7	0.69 (*p* = 0.03)	87.7 90.8	0.65 (0.40–1.05)	46.5
Bahadoer R RAPIDO NCT-01804790	2020	Phase III	912	• CRT→Sx→ CT x 6m • SCRT→XELOX x6/FOLFOX x9→Sx	50.4 25	450 462	14.3 28.4	69.6 76.3	0.75 (0.60–0.96)	88.8 89.1	0.92 (0.67–1.25)	54

CRT: chemo-radiotherapy; CT: chemotherapy; DFS: disease-free-survival; HR: hazard ratio; m: months; OS: overall survival; pCR: pathologic complete response; RT: radiotherapy; Gys: grays; SCRT: short-course radiotherapy; Sx: surgery.

**Table 2:** Patient characteristics of randomized controlled trials included.

Study	Year	*n*	Arm	T4 *n* (%)	N+ *n* (%)	N2 *n* (%)	CRM+ *n* (%)	Location		
								Low *n* (%)	Mid *n* (%)	Upper *n* (%)
Fernandez-Martos C (GCR-3)	2010	108	Std: 52 Exp: 56	3 (5.8) 7 (12.5)	NR	NR	5 (9.6) 0 (0.0)	12 (23) 18 (32)	NR	NR
Marechal R	2012	57	Std: 29 Exp: 28	3 (10.3) 2 (7.1)	25 (86.2) 26 (92.8)	NR	NR	13 (44.9) 11 (39.3)	9 (31.0) 13 (46.4)	7 (24.1) 4 (14.3)
Borg C (INOVA)	2014	91	Std: 45 Exp: 46	0 (0.0) 0 (0.0)	37 (82.2) 34 (73.9)	5 (11.1) 9 (19.6)	NR	18 (40.0) 18 (39.1)	27 (60.0) 28 (60.9)	0 (0.0) 0 (0.0)
Bujko K	2016	515	Std: 254 Exp: 261	163 (64.2) 165 (63.2)	NR	NR	NR	138 (54.3) 148 (56.7)	99 (39.0) 106 (40.6)	16 (6.3) 7 (2.7)
Moore J (WAIT)	2017	49	Std: 24 Exp: 25	1 (4.2) 5 (20.0)	22 (91.7) 25 (100)	19 (76.0) 15 (62.5)	15 (60.0) 12 (50.0)	NR		
Kim SY	2018	108	Std: 55 Exp: 53	10 (18.2) 9 (17.0)	51 (92.7) 49 (92.4)	NR	16 (29.1) 14 (26.4)	NR		
Conroy T (PRODIGE23)	2020	461	Std: 230 Exp: 231	35 (15.2) 41 (17.7)	207 (90.0) 206 (89.2)	NR	64 (27.8) 60 (26.0)	83 (36.1) 87 (37.7)	118 (51.3) 114 (49.3)	29 (12.6) 30 (13.0)
Badahoer R (RAPIDO)	2020	912	Std: 450 Exp: 462	137 (30.4) 147 (31.8)	NR	295 (65.6) 302 (65.3)	271 (60.2) 285 (60.7)	114 (25.8) 103 (22.4)	148 (33.6) 180 (39.1)	21 (4.8) 32 (6.9)
TOTAL	-	2301		728 (31.6)	682 (89.1)	645 (61.3)	742 (45.3)	763 (43.6)	842 (48.1)	146 (8.3)

CRM: circumferential resection margin; exp: experimental; n: number of patients; NR: not reported; N+: lymph nodes involved; N2: >3 lymph nodes involved; std: standard.

## Supplementary Materials

The following are available online at https://www.mdpi.com/2072-6694/12/12/3655/s1, Text S1: Search Strategy, Figure S1: Risk of bias summary of included trials organized by domain, Figure S2: Risk of bias summary of included trials organized by study, Figure S3: Funnel plots analysis of publication bias for (A) pathological complete response; (B) 3-year disease free survival; (C) 3-year overall survival, Figure S4: (A) Forest plot for R0 resection rate of TNT vs. standard neoadjuvant CRT. (B) Forest plot for tumor regression grade 3–4 rate of TNT vs. standard neoadjuvant CRT, Table S1: Surgical outcomes of randomized controlled trials included, Table S2: Sensitivity analysis for pathological complete response, Table S3: Sensitivity analysis for pathological complete response in trials using induction chemotherapy, Table S4: Sensitivity analysis for pathological complete response in trials using consolidation chemotherapy, Table S5: Sensitivity analysis for pathological complete response in trials using long-course chemoradiotherapy.
