# Supplementary material for: Impact of Total Neoadjuvant Therapy vs. Standard Chemoradiotherapy in Locally Advanced Rectal Cancer: A Systematic Review and Meta-Analysis of Randomized Trials
Source: Cancers (Basel). 2020 Dec 5;12(12):3655. doi: 10.3390/cancers12123655 (PMC7762140; doi:10.3390/cancers12123655)
Supplement: Supplementary file 1 [file cancers-12-03655-s001.pdf]

Supplementary Materials

Impact of Total Neoadjuvant Therapy vs Standard Chemoradiotherapy in Locally Advanced Rectal Cancer: A Systematic Review and Meta-Analysis of Randomized Trials

Maria C. Riesco-Martinez, Carlos Fernandez-Martos, Cristina Gravalos-Castro, Paula Espinosa-Olarte Paula, Anna La Salvia, Luis Robles-Diaz, Andrea Modrego-Sanchez, Rocio Garcia-Carbonero

Text S1. Search Strategy.

#1 rectal cancer [Mesh]
#2 rectal cancer [All fields]
#3 rectal neoplasm [All fields]
#4 #1OR #2 OR #3
#5 chemoradiotherapy [Mesh]
#6 chemoradiotherapy [All fields]
#7 chemoradiation [All fields]
#8 radiotherapy [Mesh]
#9 radiotherapy [All fields]
#10 neoadjuvant therapy [Mesh]
#11 neoadjuvant treatment [All fields]
#12 induction therapy [All fields]
#13 total neoadjuvant [All fields]
#14 chemotherapy [Mesh]
#15 chemotherapy [All fields]
#16 neoadjuvant chemotherapy [All fields]
#17 #5 OR #6 OR #7 OR #8 OR #9 OR #10 OR #11 OR #12 OR #13 OR #14 OR #15 OR #16
#18 randomized controlled trial [Mesh]
#19 randomized [All fields]
#20 controlled [All fields]
#21 #18 OR #19 OR #20
#22 #4 AND #17
#23 #22 AND #21

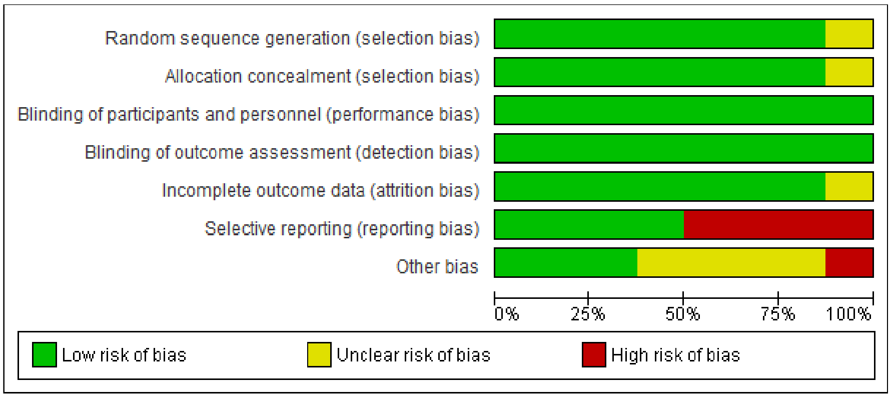

Figure S1. Risk of bias summary of included trials organized by domain.

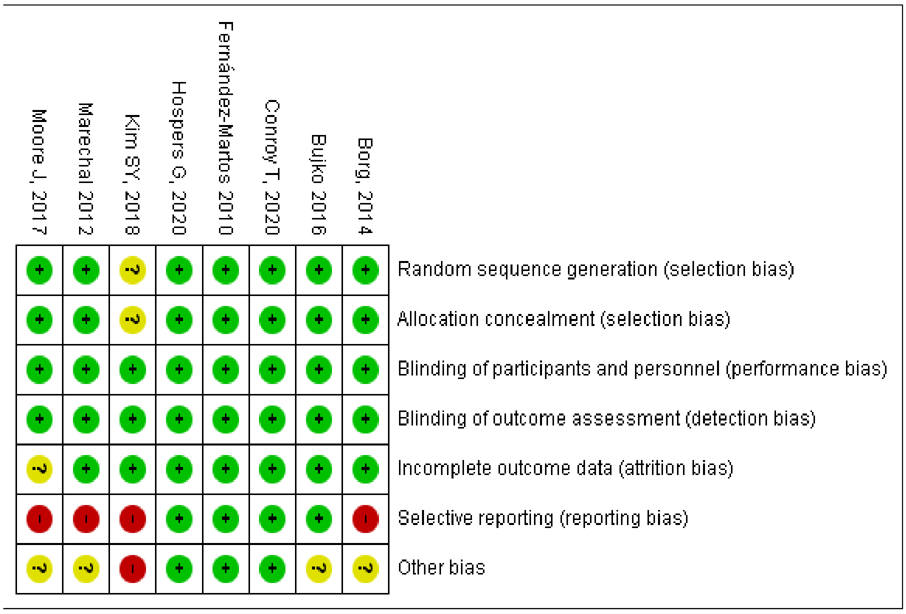

Figure S2. Risk of bias summary of included trials organized by study.

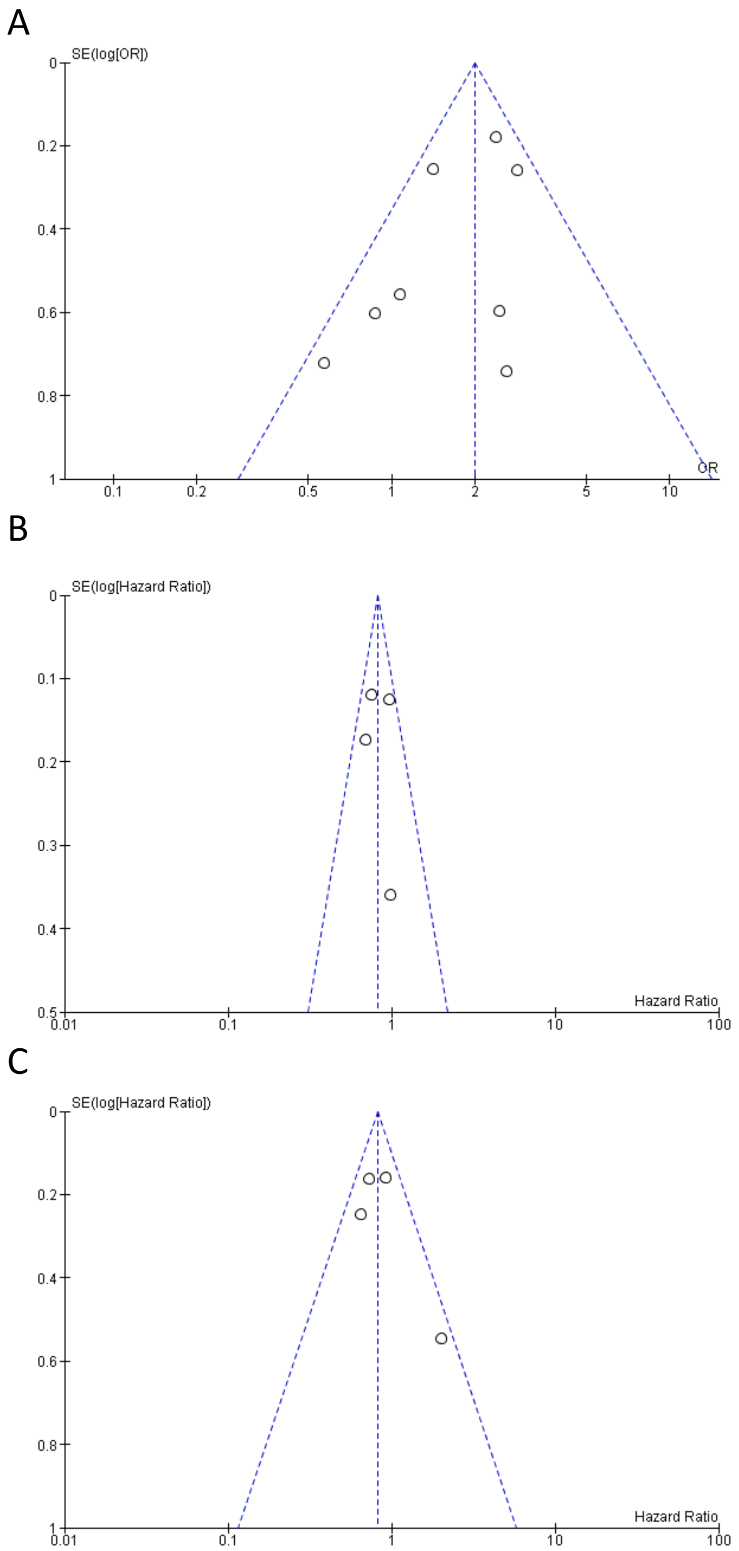

Figure S3. Funnel plots analysis of publication bias for (**A**) pathological complete response; (**B**) 3-year disease free survival; (**C**) 3-year overall survival.

A

Study or Subgroup	Experimental Events	Experimental Total	Control Events	Control Total	Weight	Odds Ratio M-H, Fixed, 95% CI	Year
Fernández-Martos 2010	48	56	45	52	7.3%	0.93 [0.31, 2.78]	2010
Marechal 2012	27	28	25	29	1.0%	4.32 [0.45, 41.31]	2012
Bujko 2016	202	261	178	254	44.7%	1.46 [0.98, 2.17]	2016
Moore J, 2017	22	24	23	25	2.1%	0.96 [0.12, 7.40]	2017
Kim SY, 2018	39	44	52	52	6.5%	0.07 [0.00, 1.27]	2018
Bahadoer R, 2020	383	423	360	398	38.5%	1.01 [0.63, 1.61]	2020
**Total (95% CI)**		**836**		**810**	**100.0%**	**1.18 [0.89, 1.55]**	
Total events	721		683				

Heterogeneity: Chi² = 6.69, df = 5 (P = 0.24); I² = 25%
Test for overall effect: Z = 1.14 (P = 0.25)

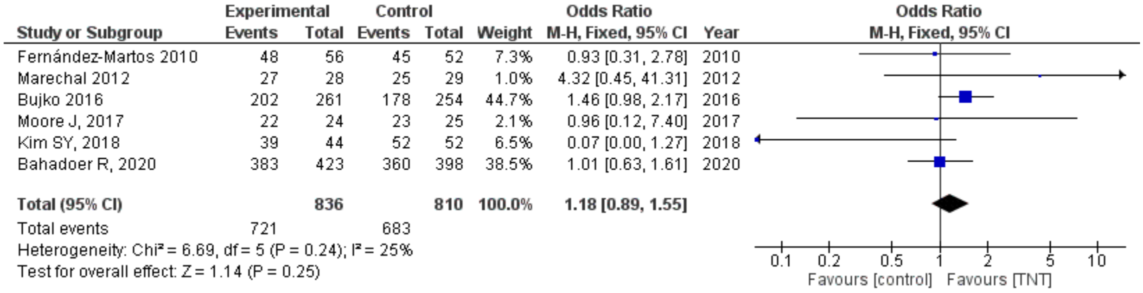

B

Study or Subgroup	Experimental Events	Experimental Total	Control Events	Control Total	Weight	Odds Ratio M-H, Fixed, 95% CI	Year
Fernández-Martos 2010	28	56	29	52	53.1%	0.79 [0.37, 1.69]	2010
Marechal 2012	13	28	13	29	24.1%	1.07 [0.38, 3.03]	2012
Kim SY, 2018	13	44	10	52	22.8%	1.76 [0.68, 4.54]	2018
**Total (95% CI)**		**128**		**133**	**100.0%**	**1.08 [0.65, 1.80]**	
Total events	54		52				

Heterogeneity: Chi² = 1.67, df = 2 (P = 0.43); I² = 0%
Test for overall effect: Z = 0.29 (P = 0.77)

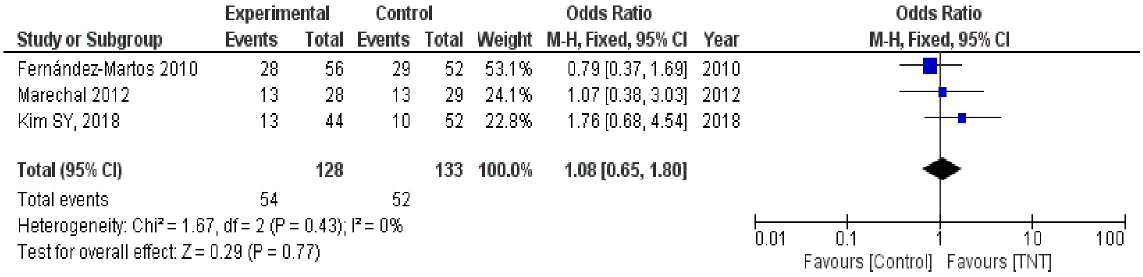

Figure S4. (**A**) Forest plot for R0 resection rate of TNT vs. standard neoadjuvant CRT. (**B**) Forest plot for tumor regression grade 3-4 rate of TNT vs. standard neoadjuvant CRT.

Table S1. Surgical Outcomes.

Study	n	Arm	Type of Surgery				Post-Operative Mortality n (%)	p
			None n (%)	Anterior Resection n (%)	APR n (%)	Missing		
Fernandez-Martos C (GCR-3)	108	Std: 52<br>Exp:56	6 (11)<br>2 (4)	29 (56)<br>27 (48)	17 (33)<br>23 (40)	0 (0)<br>2 (4)	3 (7)<br>0 (0)	ns
Marechal R	57	Std: 29<br>Exp:28	1 (3)<br>1 (4)	23 (79)<br>5 (17)	24 (86)<br>3 (11)	-	NR	
Borg C (INOVA)	91	Std: 45<br>Exp:46	NR				0(0)<br>0(0)	ns
Bujko K	515	Std: 254<br>Exp:261	49<br>41	100 (49)<br>110 (50)	83<br>82	-	4 (2)<br>0(0)	ns
Moore J (WAIT)	49	Std: 24<br>Exp: 25	0 (0)<br>0 (0)	12(50)<br>18 (72)	12 (50)<br>7 (28)	-	0(0)<br>0(0)	ns
Kim SY	108	Std:55<br>Exp:53	-	46 (88.5)<br>34 (77.3)	0 (0.0)<br>3 (6.8)	-	0(0)<br>0(0)	ns
Conroy T (PRODIGE23)	461	Std:230<br>Exp:231	NR			-	6(2.8)<br>0(0)	0.03
Badahoer R (RAPIDO)	912	Std:450<br>Exp:462	2 (0.5)<br>3 (0.7)	33 (8.3)<br>40 (9.4)	157 (39.3)<br>147 (34.5)	-	NR	

APR: abdominoperineal resection; Exp: experimental arm; n: number of patients; NR: not reported; ns: not significant; std: standard arm.

Table S2. Sensitivity analysis for pathological complete response.

Study Excluded	Fixed Effect		I^2 (%)	I^2- *p* Value
	OR	95% CI		
Fernandez-Martos C	2.04	1.62–2.57	38	0.14
Marechal	2.05	1.63–2.58	34	0.17
Borg	1.97	1.57–2.48	45	0.09
Bujko	2.17	1.68–2.79	31	0.19
Moore	2.06	1.64–2.59	24	0.24
Kim SY	1.98	1.57–2.48	45	0.09
Conroy T	1.82	1.41–2.34	30	0.20
Bahadoer R	1.75	1.30–2.35	35	0.16

CI: confidence interval; I^2: ; OR: Odds Ratio.

Table S3. Sensitivity analysis for pathological complete response in trials using induction chemotherapy.

Study Excluded	Fixed Effect		I^2 (%)	I^2- *p* Value
	OR	95% CI		
Fernández-Martos C	2.37	1.54–3.63	38	0.20
Marechal M	2.39	1.57–3.66	20	0.29
Borg C	2.10	1.38–3.20	59	0.09
Conroy T	1.32	0.69–2.53	0	0.44

CI: confidence interval; I^2: ; OR: Odds Ratio.

Table S4. Sensitivity analysis for pathological complete response in trials using consolidation chemotherapy.

Study Excluded	Fixed Effect		I^2 (%)	I^2- *p* Value
	OR	95% CI		
Bujko K	2.19	1.58–3.05	46	0.16
Moore J	2.02	1.53–2.68	32	0.23
Kim SY	1.90	1.44–2.51	65	0.06
Bahadoer R	1.36	0.87–2.12	9	0.33

CI: confidence interval; I^2: ; OR: Odds Ratio.

Table S5. Sensitivity analysis for pathological complete response in trials using long-course chemoradiotherapy.

Study Excluded	Fixed Effect		I^2 (%)	I^2- *p* Value
	OR	95% CI		
Fernández-Martos C	2.12	1.44–3.14	41	0.15
Marechal R	2.15	1.46–3.16	35	0.19
Borg C	1.92	1.31–2.82	50	0.09
Moore J	2.16	1.48–3.17	20	0.29
Kim SY	1.93	1.32–2.82	50	0.09
Conroy T	1.28	0.75–2.20	0	0.43

CI: confidence interval; I^2; OR: Odds Ratio.

© 2020 by the authors. Licensee MDPI, Basel, Switzerland. This article is an open access article distributed under the terms and conditions of the Creative Commons Attribution (CC BY) license (http://creativecommons.org/licenses/by/4.0/).
